# Clinical and echocardiographic characteristics associated with the evolution of the ductus arteriosus in the neonate with birth weight lower than 1,500g

**DOI:** 10.1590/S1679-45082013000300010

**Published:** 2013

**Authors:** Luiza Fortunato Visconti, Samira Saady Morhy, Alice D'Agostini Deutsch, Gláucia Maria Penha Tavares, Tatiana Jardim Mussi Wilberg, Felipe de Souza Rossi

**Affiliations:** 1 Hospital Israelita Albert Einstein, São Paulo, SP, Brazil

**Keywords:** Ductus arteriosus patency, Neonate, Premature

## Abstract

**Objective:**

To identify clinical and echocardiographic parameters associated with the evolution of the ductus arteriosus in neonates with birth weight lower than 1,500g.

**Methods:**

Retrospective study of 119 neonates in which clinical parameters (Prenatal: maternal age, risk of infection and chorioamnionitis, use of corticosteroid, mode of delivery and gestational age. Perinatal: weight, Apgar score, gender and birth weight/gestational age classification; Postnatal: use of surfactant, sepsis, fluid intake, heart murmur, heart rate, precordial movement and pulses, use of diuretics, oxygenation index, desaturation/apnea, ventilatory support, food intolerance, chest radiography, renal function, hemodynamic instability, and metabolic changes) and echocardiographic parameters (ductus arteriosus diameter, ductus arteriosus/weight ratio, left atrium/ aorta ratio, left ventricular diastolic diameter, and transductal flow direction, pattern and velocity) were analyzed. The clinical and echocardiographic parameters analyzed were considered statistically significant when p<0.05.

**Results:**

In the 119 neonates, the incidence of patent ductus arteriosus was 61.3%; 56 received treatment (46 pharmacological and 10 surgical treatment), 11 had spontaneous closure, 4 died, and 2 were discharged with patent ductus arteriosus. A higher incidence of chorioamnionitis, use of surfactant, lower weight and gestational age, sepsis, heart murmur, ventilatory support and worse oxygenation indices were observed in the neonates receiving treatment. The group with spontaneous closure had a smaller ductus arteriosus diameter, lower ductus arteriosus/weight ratio, and higher transductal flow velocity.

**Conclusion:**

Based on clinical and echocardiographic parameters, the neonates with spontaneous closure of the ductus arteriosus could be differentiated from those who required treatment.

## RESUMO

**Objetivo:**

Identificar parâmetros clínicos e ecocardiográficos associados à evolução do canal arterial em recém-nascidos com peso de nascimento <1.500g.

**Métodos:**

Estudo retrospectivo de 119 recém-nascidos, no qual foram analisados parâmetros clínicos (pré-natais: idade materna, risco infeccioso e corioamnionite, uso de corticoide, tipo de parto e idade gestacional; perinatais: peso, Apgar, gênero e classificação peso/idade gestacional; pós-natais: surfactante, sepse, oferta hídrica, sopro cardíaco, frequência cardíaca, movimento precordial e pulsos, diurético, índice de oxigenação, queda de saturação/apneia, suporte ventilatório, intolerância alimentar, radiografia de tórax, função renal, instabilidade hemodinâmica e alterações metabólicas); parâmetros ecocardiográficos (diâmetro do canal arterial, relação canal arterial/peso, relação átrio esquerdo/ aorta, diâmetro diastólico ventrículo esquerdo, direção, padrão e velocidade de fluxo pelo canal arterial). Os parâmetros clínicos e ecocardiográficos analisados foram considerados estatisticamente significantes quando p<0,05.

**Resultados:**

Nos 119 recém-nascidos, a incidência de canal arterial foi de 61,3%, 56 receberam tratamento (46 medicamentoso e 10 cirúrgico), 11 tiveram fechamento espontâneo, 4 foram a óbito e 2 receberam alta com persistência do canal arterial. Houve maior incidência de corioamnionite, uso de surfactante, menor peso e idade gestacional, sepse, sopro cardíaco, ventilação e piores índices de oxigenação nos recém-nascidos tratados. O grupo com fechamento espontâneo apresentou menor diâmetro do canal arterial, menor relação canal arterial/peso e maior velocidade do fluxo pelo canal arterial.

**Conclusão:**

Com base em parâmetros clínicos e ecocardiográficos, foi possível diferenciar os recém-nascidos com fechamento espontâneo do canal arterial daqueles com necessidade de tratamento.

## INTRODUCTION

The ductus arteriosus (DA) is a structure that is present in the fetus and connects the aorta and the pulmonary artery, diverting the right ventricular flow into the systemic circulation^(1,2)^.

Soon after birth, the process of functional DA closure starts^(1)^. In preterm neonates (PTN), patent ductus arteriosus (PDA) is common due to increased sensitivity to prostaglandins, a higher incidence of hypoxia and acidosis, and defective migration of the smooth muscle that leads to DA vasoconstriction^(3–5)^.

With PDA, there is high pulmonary flow and low systemic flow, which may be associated with morbidities in the PTN, such as chronic pulmonary disease, ventricular hemorrhage, leukomalacia, necrotizing enterocolitis, renal failure, congestive heart failure, retinopathy of prematurity, and increased mortality in patients with respiratory distress syndrome^(6,7)^.

For the assessment of DA, in addition to the clinical characteristics resulting from the hemodynamic changes caused by PDA, echocardiographic data that describe the cardiac morphology (DA and cardiac chamber sizes)^(8,9)^, and the flow through the DA (flow direction, pattern and velocity)^(10–13)^ are also considered.

The procedures currently available for the treatment of PDA are the use of non-steroidal antiinflammatory drugs and surgical closure. However, although these treatments have been proven effective, their use remains controversial due to their side effects (risk of transient change in brain perfusion and transient decrease in renal function when antiinflammatory drugs are used; and pneumothorax, infection, hemorrhage, chylothorax and vocal cord paralysis when surgery is used), and to the lack of evidence that they improve the patients' long-term outcome^(7)^.

Additionally, data from the literature have shown spontaneous closure occurring in the neonatal period in 31% to 34% of the neonates weighing ≤1,000g^(3,14)^ and in 67% of those weighing between 1,000 and 1,500g^(14)^, within 7 days after birth.

Thus, further studies are required to identify the groups which would really benefit from the treatment of PDA^(15,16)^.

## OBJECTIVE

To identify clinical and echocardiographic parameters associated with the evolution of the ductus arteriosus in neonates weighing <1,500g.

## METHODS

Single-center, cohort, longitudinal and retrospective study conducted in the Neonatal Intensive Care Unit of *Hospital Israelita Albert Einstein* (HIAE), in São Paulo.

The study was approved by the HIAE Research Ethics Committee no. 314/2011.

The study population comprised neonates with birth weight (BW) <1,500g, in the period from January 2008 to December 2010.

The diagnosis of PDA was made using bedside echocardiography, requested by the neonatologist.

Patients with complex congenital heart defects or those who did not undergo echocardiography were excluded from the study.

For the analysis of variables, the patients were divided into three groups: patients who had spontaneous closure of the DA (Group I); patients who received pharmaceutical treatment (Group II); and patients who received surgical treatment (Group III).

### Clinical parameters

Prenatal, perinatal and postnatal clinical parameters were analyzed. The following prenatal clinical parameters were studied: maternal age, presence of risk of infection (ruptured membranes for longer than 18 hours, maternal urinary infection, and maternal fever), presence of chorioamnionitis, antenatal use of corticosteroid, mode of delivery (vaginal or cesarean), gestational age (GA – the GA analyzed was that considered by the service, based on the last menstrual period, on the first-trimester ultrasonography, or on the Capurro's method^(17)^. The perinatal parameters analyzed were: BW, 1 and 5-minute Apgar score, gender, classification as small for GA (SGA) or appropriate for GA (AGA), based on the Babson-Benda growth charts^(18)^. The postnatal parameters observed were: use of surfactant, presence of early or late neonatal sepsis, and fluid intake in the first two and four days of life.

On the day the patients underwent echocardiographic study, in which either spontaneous closure of DA was observed or pharmaceutical or surgical treatment were indicated, the following parameters were also collected: age, presence of heart murmur, presence of persistent tachycardia (heart rate – HR>160), hyperdynamic precordium (visible *ictus*), wide pulses, use of diuretics, renal function, ventilation difficulty (as assessed by the oxygenation index), desaturation or presence of apnea, need for ventilatory support, food intolerance, chest radiography, hemodynamic instability, and metabolic changes.

### Echocardiographic parameters

The echocardiographic variables were obtained by reviewing the reports and echocardiographic images stored. Data from all echocardiograms performed were collected, from hospitalization until the test in which DA closure was verified. On the first echocardiographic study performed, the parameters described below were analyzed. In the other echocardiographic studies, only DA persistence/closure was observed.

**Figure 1:**
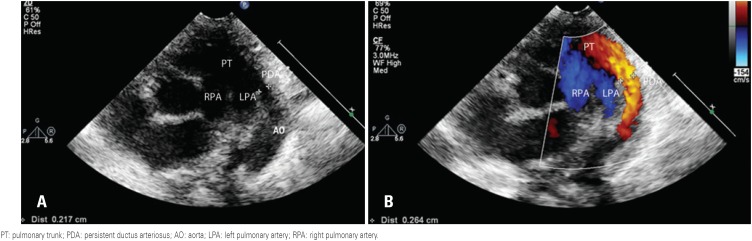
(A) Two-dimensional image showing the presence of the ductus arteriosus, with its diameter measurement. (B) Two-dimensional image of the ductus arteriosus, with color flow mapping, showing the transductal flow in red, going from the aorta to the pulmonary artery

The following variables were analyzed: DA diameter (mm) (Figure 1), DA diameter corrected for the patient's weight (mm/kg), on two-dimensional echocardiography; left atrial to aorta diameter ratio, left ventricular diastolic diameter (mm) on M-mode echocardiography; and transductal flow direction, peak velocity (m/s) and patterns on color pulsed Doppler.

**Figure 2:**
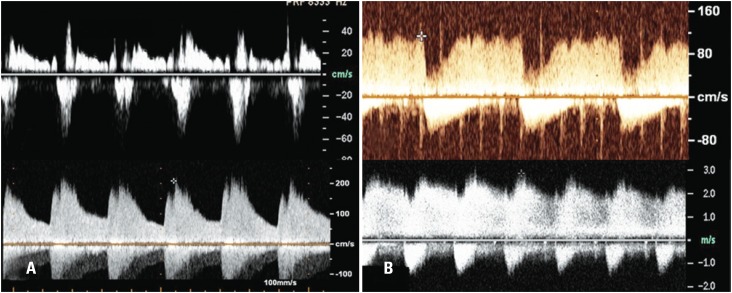
Transductal flow pattern on pulsed Doppler^(13)^. (A) Hypertension: bidirectional flow from the pulmonary to the aorta, on early systole (below baseline), followed by a small flow from the aorta to the pulmonary (above baseline). (B) Growing: bidirectional flow, however with decreased flow from the pulmonary to the aorta and increased flow from the aorta to the pulmonary (C) Pulsatile: flow only from the aorta to the pulmonary, pulsatile, with peak velocity of approximately 1.5m/s. (D) Closing: flow directed from the aorta to the pulmonary, not pulsatile but rather continuous, with a higher peak velocity

The transductal flow patterns were classified as: a) pulmonary hypertension pattern, if bidirectional flow, from the pulmonary to the aorta, in early systole (below baseline), followed by a small flow from the aorta to the pulmonary (above baseline), suggesting increased pulmonary vascular resistance; b) growing pattern, if bidirectional flow, with decreased flow from the pulmonary to the aorta, and increased flow from the aorta to the pulmonary, suggesting decreased pulmonary vascular resistance; c) pulsatile pattern, if only flow from the aorta to the pulmonary, pulsatile, with peak velocity of approximately 1.5m/s; d) closing pattern, if flow directed from the aorta to the pulmonary, not pulsatile but rather continuous, with a higher peak velocity^(13)^ (Figure 2).

### Statistical analysis

The SAS 9.0 software program was used for the statistical analyses.

Continuous variables were described as mean and standard deviation or median and interquartile range. Categorical variables were described as absolute and relative frequencies. The association between categorical variables was tested using the χ^2^ test. The Student t test was used for comparisons between numerical variables.

Results were considered statistically significant when p<0.05.

## RESULTS

In the study period, 138 patients with BW<1,500g were born. Of these, 19 were excluded, 2 for congenital heart defect (truncus arteriosus type II and coarctation of the aorta), and 17 for not having undergone echocardiography (5 died in the first day of life and the test was not requested by the medical team for the other 12). Thus 119 patients were included in the study.

The incidence of DA was 61.3% (73/119). Among neonates weighing <1,000g, the incidence was 75% (30/40), and, among those with less than 30 weeks, the incidence was 75% (57/76).

Mortality was 10.1% (12/119), of whom only one patient did not have PDA.

Of the 73 patients with PDA, 4 (5.5%) died early, before the 5^th^ day of life, with no possibility of treatment, and 2 (2.8%) were discharged with PDA, untreated.

Spontaneous closure (Group I) was observed in 11 patients (15%). The mean time for closure was approximately 13.5 days; one of these patients had spontaneous closure at 18 days, but died at 162 days of life.

A total of 46 patients (63%) underwent pharmacological treatment (Group II), with a mean closure time of 8 days of life. Of these, 3 died between the 12^th^ and 17^th^ day of life, after DA closure.

Ten patients (13.7%) underwent surgical closure (Group III), four as the first treatment choice and six after unsuccessful pharmacological treatment (mean closure time 12.7 days of life). Of these, three died at 14, 35 and 41 days of life (the time elapsed between surgery and death was longer than 1 week in the three cases).

### Clinical parameters

We observed a higher incidence of the use of surfactant in Group II in relation to Group I; a higher incidence of risk of infection, chorioamnionitis, late sepsis, and use of surfactant, as well as of lower BW, GA, and 1-minute Apgar score in Group III in relation to Group I, with statistically significant differences (p<0.05) (Table 1).

**Table 1:** Differences of pre- and perinatal variables among patients with spontaneous ductus arteriosus closure (Group I), patients with pharmacological closure (Group II) and those with surgical closure (Group III)

Patient characteristics	Group I (n=11)	Group II (n=46)	Group III (n=10)
Maternal age (years)	33.9	34.7*	38.8***
Risk of infection, n (%)	1 (9.1)	15 (34.09)*	6 (60.0%)****
Chorioamnionitis, n (%)	1 (9.1)	9 (20.0)*	6 (66.7)****
Antenatal use of corticosteroid, n (%)	9 (81.8)	29 (63.0)*	10 (100)***
Cesarean delivery, n (%)	9 (81.8)	38 (82.61)*	8 (80.0)***
Gestational age (weeks)	29.6	28.4*	25.9****
Birth weight (grams)	1.154.1	1.092.7*	828.5****
1-minute Apgar score	7.6	6.6*	4.8****
5-minute Apgar score	8.5	8.6*	8.2***
Male gender, n (%)	5 (45.5)	29 (63.04)*	8 (80)***
Classification as SGA, n (%)	3 (27.3)	8 (17.39)*	1 (10)***
Use of surfactant, n (%)	4 (36.4)	34 (73.9)**	8 (80)****
Early sepsis, n (%)	5 (45.5)	27 (58.7)*	8 (80)***
Late sepsis, n (%)	4 (36.4)	16 (34.8)*	8 (80)****
FI at 48 hours	96.5	93.6*	102.6***
FI at 96 hours	112.2	108.4*	126.4***

* No statistically significant difference was observed between Groups I and II;

** statistically significant difference was observed between Groups I and II, p<0.05;

*** no statistically significant difference was observed between Groups I and III;

**** statistically significant difference was observed between Groups I and III, p<0.05.

SGA: small for gestational age; FI: fluid intake.

A higher incidence of heart murmur and need for ventilatory support was observed in Groups II and III. Values of oxygenation index and metabolic changes were only assessed in patients with blood gas analysis on the day the treatment was indicated or spontaneous DA closure occurred; few Group I patients had their blood gas collected – this explains the difference between the two groups (Table 2).

**Table 2:** Differences in clinical signs among patients with spontaneous ductus arteriosus closure (Group I), patients with pharmacological closure (Group II) and those with surgical closure (Group III)

Clinical signs		Group I (n=11)	Group II (n=46)	Group III (n=10)
Patient age (days)		2.5	2.2*	2.1***
Presence of heart murmur, n (%)		2 (18.2)	22 (47.8)**	9 (90)****
HR >160, n (%)		0 (0)	1 (2.2)*	1 (10)***
Hyperdynamic precordium, n (%)		0 (0)	4 (40)*	2 (66.7)***
Wide pulse, n (%)		1 (50)	8 (61.5)*	2 (100)***
Use of diuretics, n (%)		0 (0)	1 (2.2)*	1 (10)***
Abnormal renal function, n (%)		1 (9.1)	0 (0)**	4 (40)***
Oxygenation index			**	****
	≤6, n (%)	0 (0)	27 (96.4)	9 (90)
	7-14, n (%)	1 (50)	1 (3.6)	1 (10)
	≥15, n (%)	1 (50)	0 (0)	0 (0)
Desaturation/apnea			*	***
	Absent, n (%)	8 (72.7)	23 (52.3)	5 (50)
	Up to 6 episodes, n (%)	2 (18.2)	19 (43.1)	2 (20)
	Frequent, n (%)	1 (9.1)	2 (4.6)	3 (30)
Ventilatory support			**	****
	Room air, n (%)	6 (54.6)	5 (10.9)	0 (0)
	Inhaled O2, n (%)	0 (0)	0 (0)	1 (10)
	CPAP ou MAP ≤8, n (%)	2 (18.2)	31 (67.4)	5 (50)
	MAP 9-12, n (%)	0 (0)	4 (8.7)	1 (10)
	MAP ≥12 HFV, n (%)	3 (27.3)	6 (13)	3 (30)
Food intolerance			*	***
	Absent, n (%)	8 (72.7)	16 (34.8)	2 (20)
	Fasting, n (%)	2 (18.2)	18 (39.1)	4 (40)
	Minimal enteral nutrition, n (%)	0 (0)	6 (13)	0 (0)
	Residual gastric content, n (%)	1 (9.1)	5 (10.9)	2 (20)
	Abdominal distention/vomiting, n (%)	0 (0)	1 (2.1)	2 (20)
Chest radiography			*	***
	Normal, n (%)	2 (50)	5 (17.2)	1 (14.3)
	Increased pulmonary vasculature, n (%)	2 (50)	20 (69)	4 (57.1)
	Cardiomegaly/pulmonary edema, n (%)	0 (0)	4 (13.8)	2 (28.6)
Hemodynamic instability			*	***
	Absent, n (%)	8 (72.7)	39 (84.8)	5 (50)
	Hypotension (1 VAD), n (%)	1 (9.1)	6 (13.0)	4 (40)
	Hypotension (more than 1 VAD), n (%)	2 (18.2)	1 (2.2)	1 (10)
Metabolic change			**	***
	Absent, n (%)	1 (33.3)	26 (92.9)	8 (80)
	Mild metabolic acidosis, n (%) (pH 7.1-7.25 and/or BE −7 to −12), n (%)	2 (66.7)	2 (7.1)	1 (10)
	Moderate to severe metabolic acidosis, n (%) (pH <7.1 or BE <-12), n(%)	0 (0)	0 (0)	1 (10)

* No statistically significant difference was observed between Groups I and II;

** statistically significant difference was observed between Groups I and II, p<0.05;

*** No statistically significant difference was observed between Groups I and III;

**** statistically significant difference was observed between Groups I and III, p<0.05.

HR: heart rate; CPAP: constant positive airway pressure; MAP: medium airway pressure; HFV: high-frequency ventilation; VAD: vasoactive drug; BE: base excess.

### Echocardiographic parameters

The echocardiographic measurements studied were compared among the three groups. The mean DA diameter and the mean weight-corrected DA diameter were greater in Groups II and III in relation to Group I. The transductal flow velocity was significantly lower in Group III in relation to Group I (Table 3).

**Table 3:** Differences of the echocardiographic measurements among patients with spontaneous ductus arteriosus closure (Group I), patients with pharmacological closure (Group II) and those with surgical closure (Group III)

Echocardiographic data	Group I (n=11)	Group II (n=46)	Group III (n=10)
DA diameter (mm)	1.63	2.24**	2.39****
DA/W (mm/kg)	1.55	2.22**	3.44****
LA/Ao	1.10	1.20*	1.26***
LV diastolic diameter (mm)	12.73	12.98*	11.78***
Flow direction (L-R), n (%)	6 (66.7)	33 (71.7)*	5 (50)***
Transductal flow velocity (m/s)	2.45	1.92*	1.62****

DA: ductus arteriosus diameter; DA/W: ductus arteriosus diameter/patient weight ratio; LA/Ao: Left atrium size/aorta size ratio; LV: left ventricle; L-R: from the left to the right.

* No statistically significant difference was observed between Groups I and II;

** statistically significant difference was observed between Groups I and II, p<0.05;

*** no statistically significant difference was observed between Groups I and III;

**** statistically significant difference was observed between Groups I and III, p<0.05.

In relation to the transductal flow patterns assessed in the first echocardiogram, 57.2% of patients with PDA with spontaneous closure had a hypertensive (14.3%) or closing pattern (42.9%), whereas 67.5% of patients undergoing pharmacological closure had a growing pattern (17.5%) or pulsatile pattern (50%), and 66.6% of patients undergoing surgical closure had a growing pattern (22.2%) or pulsatile pattern (44.4%). However, no statistically significant difference was observed between the groups (Table 4).

**Table 4:** Differences of the transductal flow patterns among patients with spontaneous ductus arteriosus closure (Group I), patients with pharmacological closure (Group II) and those with surgical closure (Group III)

Flow patterns on the first echo-cardiogram	Group I (n=11)	Group II (n=46)	Group III (n=10)
Hypertension or closing, n (%)	4 (57.2)	13 (32.5)*	3 (33.3)***
Growing or pulsatile, n (%)	3 (42.8)	27 (67.5)*	6 (66.7)***

* No statistically significant difference was observed between Groups I and II;

*** no statistically significant difference between Groups I and III.

## DISCUSSION

PDA is an anomaly that affects a large number of premature neonates and may lead to significant clinical consequences, with an impact in the morbidity and mortality of these patients.

In this study, we observed an incidence of 61.3% of PDA, of which 75% in neonates weighing <1,000g or with less than 30 weeks. This results is in agreement with data from the literature that describe incidences between 20% and 60%, depending on the diagnostic criteria and population studied, with increasing incidences with the decrease in BW and GA^(3)^.

Currently, the core issue in the clinical management of patients with PDA is to identify, by means of epidemiological, clinical and echocardiographic data, the group that would benefit from treatment indication, given the adverse consequences of the pharmacological and surgical treatments, of the incidence of spontaneous closure, and of the lack of evidence that the prophylactic pharmacological treatment is beneficial in the long term^(19–21)^.

Disagreement is common between clinical and echocardiographic parameters in the assessment of the hemodynamic consequences of PDA. In practice, DA size on echocardiogram, the presence of chronic pulmonary disease, and the increase in ventilatory support parameters are taken into consideration.

Echocardiogram can provide an early diagnosis and evaluate the hemodynamic consequences. However, there is still no consensus on which echocardiographic parameters should be used to guide therapy.

In the present study, among pre- and perinatal parameters, the use of surfactant was significantly more frequent in the groups with pharmacological and surgical closure in relation to the group with spontaneous closure, so this population could be more prone to require treatment. Surfactants have no effect on the contractile behavior of DA, but they change the pulmonary vascular resistance, increasing the left to right transductal flow^(22)^.

The presence of risk of infection, chorioamnionitis and late sepsis was more frequent in the group requiring surgery, and this could be explained by the release of inflammatory mediators in these processes; these mediators would impair the spontaneous DA closure, as described by Shannon et al.^(1)^. BW and GA were lower in the group with surgical closure, with a statistically significant difference. Jhaveri et al. also found failure in the treatment with indomethacin in 70% of neonates <28 weeks of GA, thus requiring surgical treatment^(23)^.

In relation to the echocardiographic parameters analyzed, the comparison between the three groups with PDA showed higher mean DA diameter and DA diameter/weight ratio in the groups with pharmacological and surgical closure. These results are similar to those described by Hajjar et al.^(8)^, who observed an inverse relation between DA size and the probability of spontaneous closure. Mc Namara and Sehgal also related the DA size to the hemodynamic consequences of PDA, categorizing them as small, when DA diameter is <1.5mm; moderate, when DA diameter is between 1.5 and 3mm; and large, when DA diameter >3mm^(15)^.

On the other hand, Jhaveri et al. considered small DA those with a diameter <1.5mm. However, diameters >1.5mm were considered as DA with moderate hemodynamic consequences. The difference between moderate and severe hemodynamic consequences was defined by the mean transductal flow gradient, which was <8mmHg in cases with severe consequences^(23)^.

In this study, the transductal flow velocity was higher in the cases of PDA in closing process, and lower in the group with surgical closure; these findings are similar to those observed by Su et al.^(13)^. Transductal flow acceleration in the neonate is an indirect sign of its closing process and should be taken into consideration for the decision-making concerning therapy.

However, in this study, unlike in Su et al's study^(13)^, no statistically significant difference in the transductal flow pattern was observed between the groups. In our opinion, this difference can be explained by the fact that, in this study, the flow patterns analyzed were only those from the first echocardiogram, which was performed early (mean time of 2.3 days). It would interesting to analyze follow-up echocardiographic studies to observe whether a significant difference would occur that could help decide therapy.

The major limitation of this study is that it is a retrospective study, with the patients being divided into groups according to the absence, presence or treatment of PDA, based on criteria of the neonatal intensive care unit, and data collected by means of review of medical charts and echocardiograms previously performed. Addittionally, it was not possible to evaluate all the echocardiographic parameters used to quantify the hemodynamic consequences of PDA, as reported in studies of the literature, such as: mean flow velocity in the left pulmonary artery, end-diastolic velocity in the left pulmonary artery, E/A wave ratio through the diastolic tricuspid valve flow, and reverse diastolic flow in the descending aorta and mesenteric artery^(8,15,23)^.

## CONCLUSION

In this population of neonates weighing <1,500g, the use of surfactant, presence of heart murmur, need for ventilatory support, DA diameter, and DA diameter/ weight ratio could differentiate patients who progressed with spontaneous DA closure (Group I) from those requiring pharmacological treatment (Group II) and/or surgical treatment (Group III).

Other parameters, such as risk of infection, chorioamnionitis, birth weight, gestational age, 1-minute Apgar score, presence of late sepsis, and transductal flow velocity on Doppler, could differentiate patients who progressed with spontaneous DA closure (Group I) from those requiring surgical treatment (Group III).
